# Comparative Study of the Antimicrobial Effects of Tungsten Nanoparticles and Tungsten Nanocomposite Fibres on Hospital Acquired Bacterial and Viral Pathogens

**DOI:** 10.3390/nano10061017

**Published:** 2020-05-26

**Authors:** Rupy Kaur Matharu, Lena Ciric, Guogang Ren, Mohan Edirisinghe

**Affiliations:** 1 Department of Mechanical Engineering, University College London, Torrington Place, London WC1E 7JE, UK; rupy.matharu.15@ucl.ac.uk; 2 Department of Civil, Environmental & Geomatic Engineering, University College London, Chadwick Building, Gower Street, London WC1E 6BT, UK; l.ciric@ucl.ac.uk; 3 School of Engineering and Technology, University of Hertfordshire, Hatfield AL10 9AB, UK; g.g.ren@herts.ac.uk

**Keywords:** tungsten, nanoparticles, nosocomial infection, antimicrobial, tungsten oxide, bacteria, virus

## Abstract

A significant proportion of patients acquire hospital associated infections as a result of care within the NHS each year. Numerous antimicrobial strategies, such as antibiotics and surface modifications to medical facilities and instruments, have been devised in an attempt to reduce the incidence of nosocomial infections, but most have been proven unsuccessful and unsustainable due to antibiotic resistance. Therefore, the need to discover novel materials that can combat pathogenic microorganisms is ongoing. Novel technologies, such as the potential use of nanomaterials and nanocomposites, hold promise for reducing these infections in the fight against antimicrobial resistance. In this study, the antimicrobial activity of tungsten, tungsten carbide and tungsten oxide nanoparticles were tested against *Escherichia coli*, *Staphylococcus aureus* and bacteriophage T4 (DNA virus). The most potent nanoparticles, tungsten oxide, were incorporated into polymeric fibres using pressurised gyration and characterised using scanning electron microscopy and energy dispersive X-ray spectroscopy. The antimicrobial activity of tungsten oxide/polymer nanocomposite fibres was also studied. The results suggest the materials in this study promote mediation of the inhibition of microbial growth in suspension.

## 1. Introduction

Nosocomial infections, commonly known as healthcare-acquired infections (HCAI), arise either as the direct result of healthcare interventions such as medical or surgical treatment, or from interacting with healthcare settings, i.e., nursing homes or rehabilitation facilities [1]. Such infections are spread by various means, including contaminated beds and equipment as well as through staff [2]. HCAIs are considered detrimental to patients, employees and guests and contribute significantly to patient morbidity and mortality, consequently accumulating an economical burden on the National Health Service (NHS). As a result, infection prevention and control are a priority.

The term HCAIs covers a wide range of infections with *Escherichia coli*, *Staphylococcus aureus, Pseudomonas aeruginosa* and adenovirus (a DNA virus) being amongst the leading causes [3,4]. *E. coli* alone killed more than 5500 NHS patients in 2015 [5]. In fact, The National Institute for Health and Care Excellence has estimated that 300,000 patients a year in England acquire HCAI as a result of care within the NHS and cost the NHS in excess of £1 billion annually [6]. Unfortunately, treating these common pathogenic microorganisms has become increasingly difficult as they develop mechanisms for antimicrobial resistance [7]. In turn, this facilitates the rapid spread of drug resistant microorganisms and causes traditional antimicrobial agents to become less effective [8,9]. Coupled to this, the improper use of these agents has further escalated the problem. The accelerated discovery and development of novel alternative antimicrobials and technologies is much needed in order to prevent and control infection. This paramount challenge is of utmost importance and has been addressed in the UK Five Year Action Plan for Antimicrobial Resistance 2019–2024 and the UK Twenty Year Vision for Antimicrobial Resistance.

Recent advances in nanomaterials research have encouraged the use of nanoparticles as viable solutions to ongoing problems such as uncovering new antimicrobial agents. Over the last decade there has been a tremendous growth of research activities to explore the antimicrobial properties of a variety nanoparticles, including spheres, platelets, nanorods, nanoflowers and nanobars [10,11,12,13,14,15,16,17,18,19,20]. Particles in the nanometre range exhibit unique physical and chemical properties and have been established as an increasingly important material in the development of antimicrobial agents. A number of recent studies have demonstrated the biocidal activity of nano-metal and metal oxides, as well as carbonaceous nanomaterials [15,16,21,22]. However, research into nanosized tungsten and its derivatives is scarce.

Tungsten is a d-block transition metal and holds an atomic number of 74. It can be found in its pure form, or as tungsten oxide and tungsten carbide. This oxyanion has similarities to vanadate and molybdenum and studies have shown tungsten to hold a biological function in some prokaryotes. In these organisms, tungstoenzymes, such as formate dehydrogenase, formyl methanufuran dehydrogenase, acetylene hydratase and a class of genetically related oxidoreductases, use tungsten as a tungsten-pterin to catalyse the reversible reduction of carboxylic acids to aldehydes [23,24]. The exploration of tungsten’s antiviral and antibacterial properties has stemmed from the Ren et al. patent [25] claiming the viricidal efficacy of tungsten nanoparticles, especially in combinations with other effective antimicrobial components. In this work, tungsten carbide was shown to give rise to a 99.996% reduction of avian H5N1 Influenza NIBRG-14 virus after a treatment time of 30 min. However, the antibacterial activity of tungsten and its derivatives is rarely reported in the scientific literature [26,27].

In this research, the antimicrobial activity and the minimum inhibitory concentration (MIC) of tungsten, tungsten oxide and tungsten carbide against three common HCAI pathogens (*E. coli*, *S. aureus* and a DNA viral model organism) is described. The incorporation of the most potent agent into polymeric fibres is also explored and the antimicrobial activity of the resulting fibres is determined. Dispersing the nanoparticles in a polymer matrix is an extremely attractive method not only to improve the usability of the antimicrobial agents, but also enhance the activity of the nanoparticles. Nano-polymeric composites increase the surface area of the nanoparticle by reducing the formation of nanoparticle agglomerations, this ensures high reactivity and improves the antimicrobial properties.

## 2. Materials and Methods

### 2.1. Materials

#### 2.1.1. Powders

Tungsten (powder, particle size 10 µm), tungsten oxide (nanopowder, particle size <100 nm) and tungsten carbide (nanopowder, particle size 150–200 nm) powders were purchased from Sigma Aldrich (Gillingham, UK). After preliminary experimental findings, only tungsten oxide showed significant antimicrobial potency, therefore was investigated in nanocomposite fibres.

#### 2.1.2. Fibre Production Materials

Poly(methyl methacrylate) (PMMA) (Mw 120,000 g/mol), chloroform and tungsten oxide (nanopowder, particle size <100 nm) obtained from Sigma Aldrich (Gillingham, UK) were used for preparation of fibres according to our previously published standardised method [16,28,29]. All reagents were of analytical grade and used as received.

#### 2.1.3. Microbial Strains and Media

*Escherichia coli* K12, *Staphylococcus aureus* ATCC 6538P and *Escherichia coli* bacteriophage T4 ATCC 11303-TB4 (DNA virus) were used in this study. Freeze-dried cultures of all strains were sourced from LGC Standards (Teddington, UK) and cultured following the manufacturers’ instructions. Luria Bertani (LB) broth was purchased from Invitrogen (Paisley, UK) whilst LB agar, nutrient agar, nutrient broth, phosphate buffer saline (PBS) and sodium chloride was obtained from Sigma Aldrich (Gillingham, UK). A LIVE/DEAD BacLight Bacterial Viability and Counting Kit was acquired from ThermoFisher (Loughborough, UK).

Stock cultures of *E. coli* and *S. aureus* were stored in vials in a Microbank^TM^ at −80 °C. Each stock solution was streaked onto LB agar using a sterile loop and incubated at 37 °C for 24 h, when needed. Cultures of *E. coli* ATCC 11,303 were also stored in a Microbank^TM^, but at −20 °C. Escherichia virus T4 was kept at 2 °C. This virus was selected as a representative microorganism for DNA viruses, as DNA viruses represent a large proportion of hospital acquired viruses and this strain is commonly available and safe to work with in Biosafety Level 2 laboratories.

### 2.2. Methods

#### 2.2.1. Minimum Inhibitory Concentration

Bacterial suspensions were grown in 50 mL centrifuge tubes, by inoculating 30 mL of sterile LB broth with a single bacterial colony. The suspensions were cultured at 37 °C and 150 rpm (Orbital Shaker S150, Stuart, Staffordshire, UK) until they reached their mid exponential phase (approximately 3 h). This bacterial suspension was then added to sterile LB broth containing an antimicrobial treatment (0.5, 1.0 and 2.0% wt/v of nanoparticles or 1% wt/v of PMMA nanocomposite fibres containing different tungsten oxide concentrations 0, 2, 4 and 8 wt %) at 10% v/v. The suspensions were incubated for 24 h at 37 °C and 150 rpm (Orbital Shaker S150, Stuart, Staffordshire, UK) without the addition of carbon dioxide. After incubation, the samples were vortexed for 10 s and flow cytometry was used in conjunction with the LIVE/DEAD BacLight Bacterial Viability and Counting Kit (ThermoFisher, Loughborough, UK) to quantify the antibacterial activity of the treatments tested [30]. The kit consisted of two fluorescent dyes, propidium iodide and SYTO^®^9. Viable bacteria stain green with SYTO^®^9. In non-viable cells propidium iodide displaces the SYTO^®^9 resulting in a red colouration [30]. Cell populations were gated accordingly using positive, negative, fluorescent minus one and double stained control samples.

Antiviral activity was determined using a plaque assay. An actively growing broth culture of *E. coli* 11,303 was prepared by incubating a single colony in 30 mL of sterile ATCC Medium 129 broth for 18 h at 37 °C and 150 rpm. Bacteriophage T4 suspensions containing 0.5, 1.0 and 2.0% wt/v of nanoparticles or 1% wt/v of tungsten oxide/PMMA nanocomposite fibres were prepared. Of these suspensions 100 µL at 0 and 24 h were added to 300 µL of *E. coli* and 3 mL of molten semi solid ATCC Medium 129 and poured onto ATCC Medium 129 agar plates. The plates were incubated for 24 h at 37 °C and the number of plaques were counted. Antiviral activity of the tungsten powders was statistically analysed and compared to the control using a one-way ANOVA with a post hoc Tukey Honest Significant Difference (HSD) test. The difference was considered significant when *p* < 0.05.

#### 2.2.2. Preparation of Antimicrobial Fibre Meshes

Polymer solutions containing tungsten oxide nanoparticles were prepared before being processed using pressurised gyration. First, nanoparticle suspensions were prepared by adding tungsten oxide nanoparticles to chloroform as described in Table 1. The suspensions were sonicated (S800, Branson Ultrasonics, Danbury, CT, USA) for 2 h to achieve a homogenous dispersion. Second, polymer solutions were prepared by dissolving PMMA in chloroform. The solution was mechanically stirred until completely dissolved. Finally, the tungsten oxide suspension was then combined with the polymer solution and allowed to stir for 1 h on a magnetic stirrer before being subjected to pressurised gyration [31].

The nanoparticle/PMMA solutions were processed into fibres using pressurised gyration [31]. The experimental setup consisted of a perforated rotating aluminium cylindrical pot (60 mm by 35 mm, with 24 circular orifices along its central horizontal axis) attached to a high-speed rotary motor and a nitrogen gas supply [31]. Of the nanoparticle/PMMA solution 3 mL aliquots were loaded into the pot and the pot was sealed. The system was immediately switched on and allowed to reach maximum speed before applying 0.1 MPa of pressure. The system was spun until all the solution had been ejected from the pot (approximately 1 min). Pressurised gyration experiments were conducted at a relative humidity of 55% ± 4% and an average temperature of 21 ± 2 °C. All fibres were prepared in triplicate.

#### 2.2.3. Fibre Characterisation

Fibre morphology was characterised using SEM and IMAGE J software (National Institutes of Health, Bethesda, MD, USA). The fibres were sputter-coated with gold (Q150R ES, Quorum Technologies, Lewes, UK) for 180 s prior to being imaged by SEM (JEOL JSM-6301F or FEI Inspect-F SEM). Average fibre diameter was calculated by measuring the width of 100 fibres inn IMAGE J.

Chemical composition of the fibres was assessed using energy-dispersive X-ray spectroscopy (EDX) and performed using INCA X-Sight (Oxford Instruments, Abingdon, UK). The voltage used was 20 kV and the working distance was 10 mm. INCA software (ETAS, Derby, UK) was used to analyse the EDX spectra.

#### 2.2.4. Fluorescent Microscopy of Nanocomposite Fibres

Post incubation, the fibres containing 8 wt % of tungsten oxide were functionalised with fluorescent dyes, propidium iodide and SYTO^®^9, and visualised using fluorescent microscopy (EVOS FL Cell Imaging System, Invitrogen, ThermoFisher, Loughborough, UK) equipped with a Green Fluorescent Protein (GFP) light cube (470/22 nm excitation; 510/42 nm emission) and Red Fluorescent Protein (RFP) light cube (531/40 nm excitation; 593/40 nm emission). These light cubes were selected according to the manufacturers’ guidance and the excitation/emission maxima of the fluorescent dyes. The right choice of light cube helped minimise bleed-through and ensured the right fluorophore was observed. Higher magnification images were captured using 40× objective lens.

## 3. Results

### 3.1. Nanoparticle Characterisation

**Figure 1:**
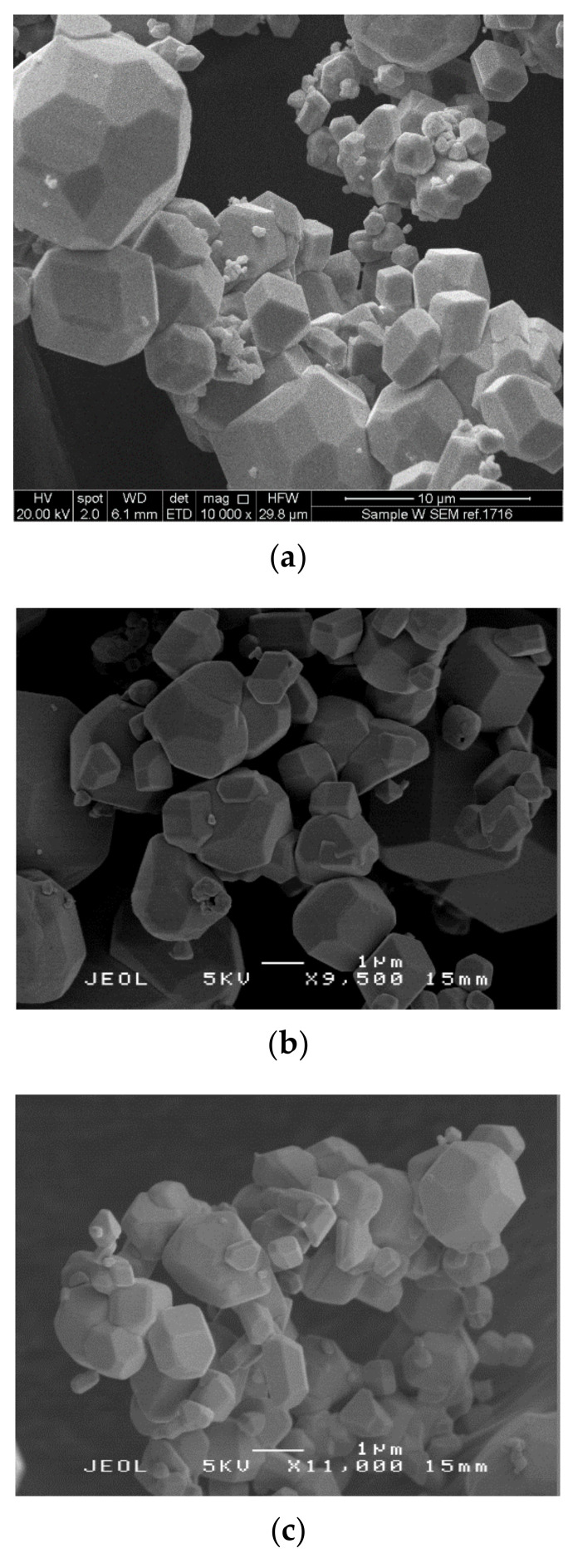
Scanning electron micrographs of (**a**) tungsten, (**b**) tungsten oxide and (**c**) tungsten carbide nanoparticles.

The nanoparticles investigated in this study were characterised using a variety of techniques to gather information on their size, shape and morphological parameters. Scanning electron micrographs of the nanomaterials are shown in Figure 1.

Micrographs of tungsten, tungsten oxide and tungsten carbide showed the particles to have polyhedron geometry. Tungsten had an average particle size of 3.0 ± 2.6 µm, tungsten oxide 1.7 ± 0.9 µm and tungsten carbide 1.6 ± 0.7 µm. It should be noted the size of the tungsten, tungsten oxide and tungsten carbide particles differ to the specification provided by the manufacturers, although they may be agglomerated.

### 3.2. Minimum Inhibitory Concentration (MIC)

**Figure 2:**
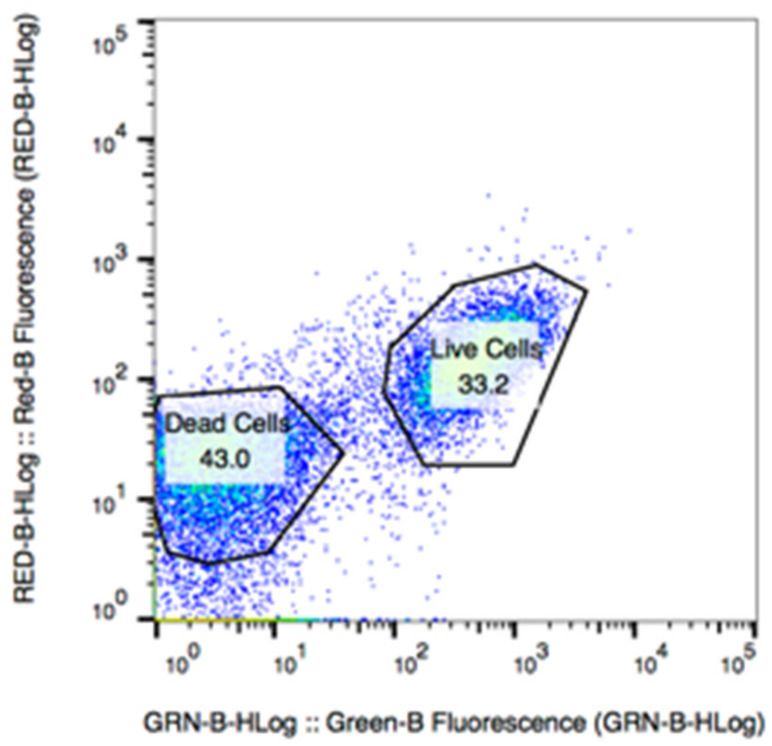
Raw flow cytometry data collected from the incubation of 0.5 wt % of tungsten oxide with *Staphylococcus aureus* cells for 24 h at 37 °C and 150 rpm.

*E. coli* K12 and *S. aureus* were used as model bacteria to evaluate the MIC of tungsten nanoparticles. A LIVE/DEAD assay was used with flow cytometry following our previously reported method [30] to assess the proportion of live and dead cells in the suspension (Figure 2).

**Figure 3:**
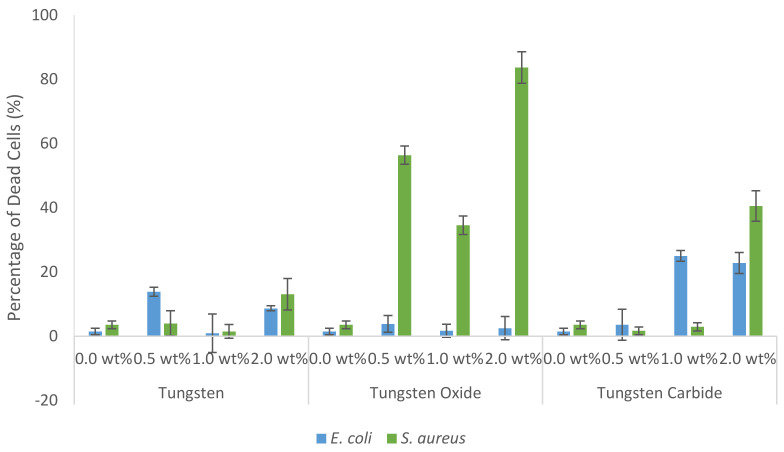
Graph showing the proportion of dead bacterial cells after 24 h incubation with 0.5, 1.0 and 2.0 wt % of tungsten, tungsten oxide and tungsten carbide. 0.0 wt % represents the untreated control group. Results gathered using flow cytometry in conjunction with LIVE/DEAD BacLight Bacterial Viability and Counting Kit. Error bars represent standard deviation, *n* = 3.

As seen in Figure 3 tungsten oxide showed the strongest antibacterial activity towards *S. aureus* with 2.0 wt % resulting in the death of 83.7% of the bacterial population. From the results gathered with *E. coli* it can be said that tungsten carbide showed moderate toxicity, with 2.0 wt % leading to the death of 22.8% of the cells. However, this level of toxicity is not strong enough to deem the material as antibacterial at this concentration. Tungsten showed no significant antibacterial effects against both bacteria tested.

**Figure 4:**
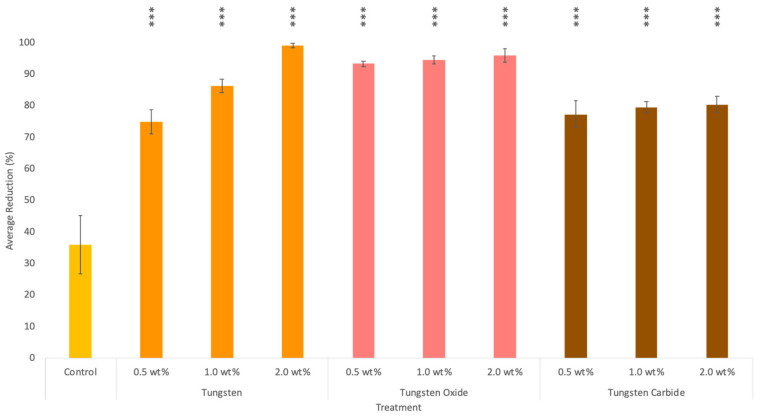
Graph showing the antiviral activity of tungsten, tungsten oxide and tungsten carbide at 0.5, 1.0 and 2.0 wt % against bacteriophage T4 for 24 h. Results were collected using a plaque assay, viral reductions were calculated from the number of virions present before and after exposure. Error bars represent standard deviation, *n* = 3. *p* values of <0.001 (***) are shown on the graph.

As seen in Figure 4 tungsten and its derivates showed dose dependent activity and stronger potency towards viruses. This finding is in corroboration with Ren et al.’s earlier observations [25]. Tungsten oxide demonstrated high overall antiviral activity, with viral reductions ranging between 93.4% and 96.0%. While tungsten exhibited antiviral properties at 2.0 wt % with a viral reduction of 99.2%.

Tungsten oxide showed the overall strongest antimicrobial activity towards both bacteria and viruses with 2.0 wt % resulting in the death of 83.7% of the bacterial population and 96.0% of the viral population. The one-way ANOVA showed an overall significant difference for all treatments (F-statistic = 71.1008, *p*-value = 3.2441●e^−13^). The post hoc Tukey HSD results showed that all treatments, when compared to the control, were statistically significant (Tukey HSD *p*-value = 0.001 and Tukey HSD interference = *p* < 0.01).

### 3.3. Characterisation of Nanocomposite Fibres

**Figure 5:**
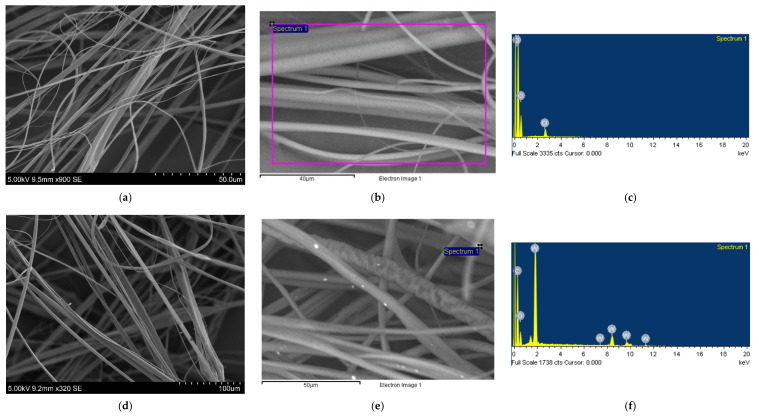

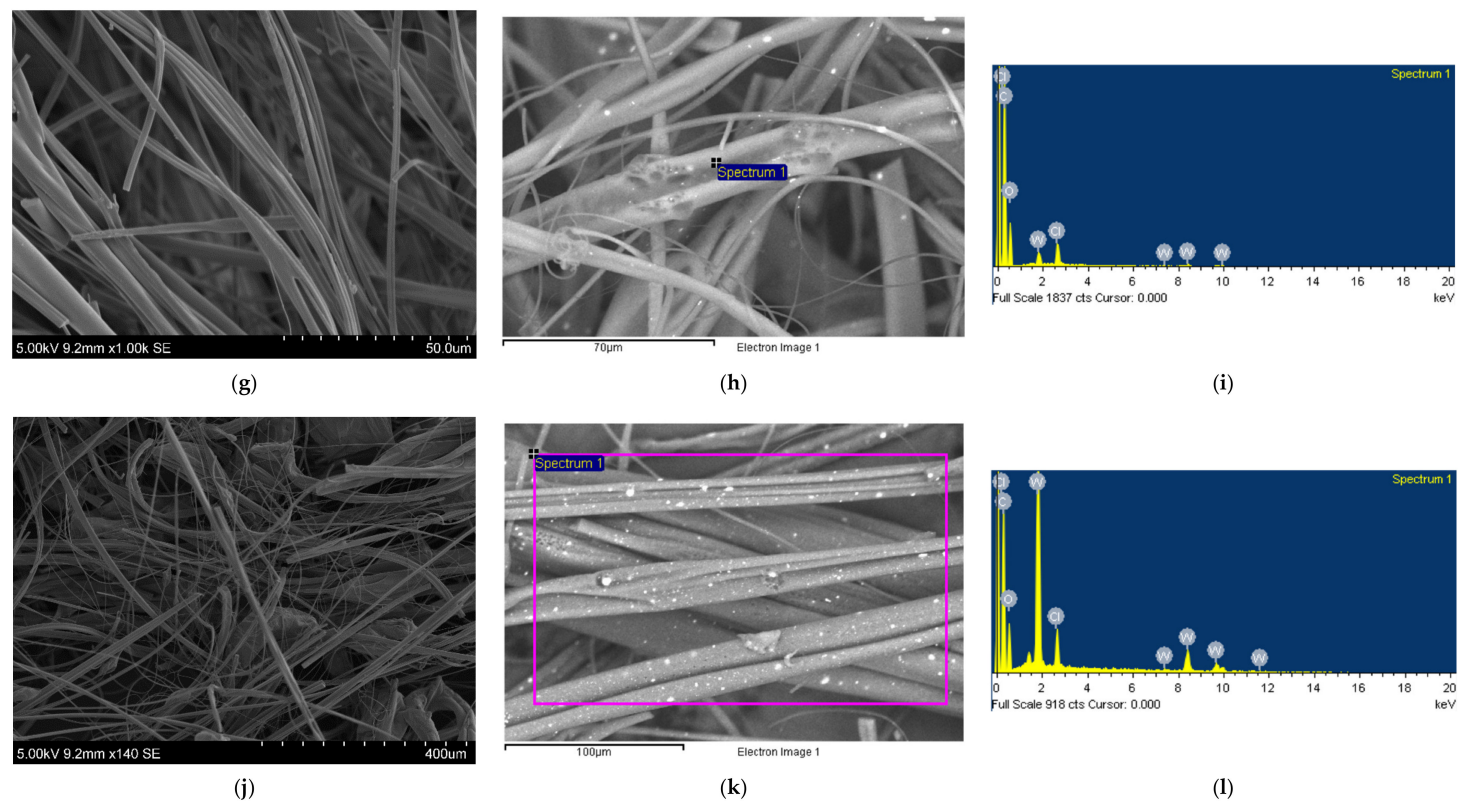
Scanning electron microscope images and EDX spectra (**a**–**c**) pure PMMA fibres; (**d**–**f**) PMMA fibres with 2 wt % of WO_3_; (**g**–**i**) PMMA fibres with 4 wt % of WO_3_ and (**j**–**l**) PMMA fibres with 8 wt % of WO_3_.

In this part of the study, tungsten oxide/polymer nanocomposite fibres were prepared with either 0, 2, 4 and 8 wt % of tungsten oxide particles located on the surface and within in the matrix. SEM images and EDX analysis of these fibres are shown in Figure 5.

For all concentrations, the fibres appeared relatively bead-free and continuous. However, fibre diameter largely depended on the tungsten oxide concentration in the resulting fibre, as a positive correlation can be observed between the two variables. Pure PMMA fibres had an average fibre diameter of 1.5 ± 1.4 µm. Fibres formed using 2 wt % of tungsten oxide had an average fibre diameter of 2.9 ± 2.1 µm, whilst fibres formed using 4 wt % of tungsten oxide had an average fibre diameter of 3.3 ± 2.0 µm. Increasing the tungsten oxide concentration to 8 wt % resulted in an average fibre diameter of 6.6 ± 4.4 µm. It was also noted that increasing the tungsten oxide concentration widened the fibre diameter distribution (provided in the supplementary information, Figure S1).

EDX imaging was used to show the successful incorporation of tungsten oxide in the fibres (Figure 5b,c,e,f,h,i,k).

### 3.4. Antimicrobial Fibre Studies

Tungsten oxide/polymer nanocomposite fibres were incubated in *S. aureus* and bacteriophage T4 suspensions (DNA virus) for 24 h at 37 °C and 150 rpm.

**Figure 6:**
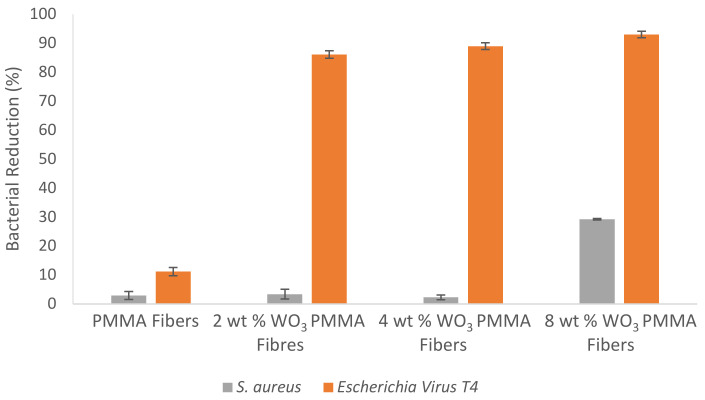
Graph showing the antimicrobial activity of tungsten oxide/polymer nanocomposite fibres against *S. aureus* and bacteriophage T4. Error bars represent standard deviation, *n* = 3.

As shown in Figure 6, pure PMMA fibres showed no antimicrobial activity with 97.1% of the bacterial population and 89.0% of the viral population remaining alive after incubation.

2 wt % and 4 wt % tungsten oxide loaded fibres also showed no antibacterial activity with 96.6% and 97.7% of the bacterial population remaining alive, respectively. The 8 wt % tungsten oxide/polymer nanocomposite fibres showed moderate antibacterial activity with 29.2% of the bacterial population dying.

The 2, 4 and 8 wt % tungsten oxide loaded fibres showed strong antiviral activity towards the DNA virus model organism, with 8 wt % demonstrating the strongest potency with a viral reduction of 93.0%.

### 3.5. Fluorescent Microscopy of Nanocomposite Fibres

**Figure 7:**
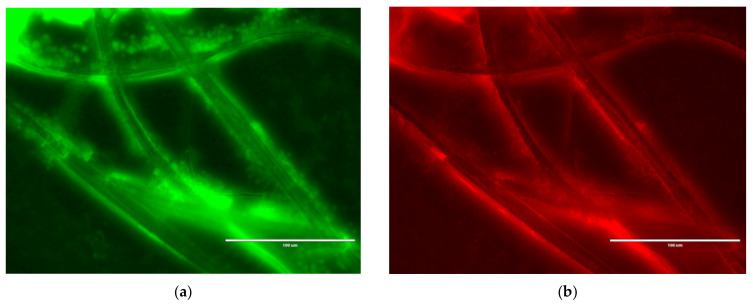
Antibacterial activity of nanocomposite fibres containing 8 wt % tungsten oxide against *S. aureus* after 24 h incubation. (**a**) Green, SYTO^®^9, corresponds to viable cells and (**b**) red, propidium iodide, corresponds to nonviable cells.

Following 24 h contact time with nanocomposite fibres containing 8 wt % of tungsten oxide, dead *S. aureus* cells can be seen on the surface of the fibres (Figure 7). Visualisation of the fibres provided verification that the microbial cells died when in contact with the fibres.

## 4. Discussion

Nanoparticles are increasingly being used as alternatives to antibiotics. However, the nanoparticles that are currently being used carry detrimental side effects if doses are too high and are ineffective against a broad spectrum of microbes, thus the discovery of new antimicrobial nanomaterials is crucial [32,33]. The utility of unexplored nanoparticles for the effective inhibition of pathogenic colonisation and proliferation is explored in this work.

### 4.1. Minimum Inhibitory Concentration

Flow cytofluorometric methods provide rapid and reproducible information on the inhibitory effect (bactericidal or bacteriostatic; time-dependent or concentration-dependent) of antimicrobial agents and the cell damage inflicted to the test microorganisms. The use of propidium iodide and SYTO^®^9 allows a clear discrimination between dead and viable cells to be made. In this study the antibacterial activity of tungsten, tungsten oxide and tungsten carbide were tested in suspension against representative Gram-negative and Gram-positive microorganisms. The viricidal properties of the nanomaterials were tested in suspension against a representative double stranded DNA virus. A plaque assay was used to quantitate the number of infectious viral particles in suspension before and after treatment. The advantage of using plaque assays is their ability to give a direct quantitative measurement of the exact number of virions in suspension [34,35].

From the results collated tungsten oxide was found to be the most effective against *S. aureus* and bacteriophage T4. The antimicrobial properties of tungsten oxide are rarely reported in the literature, despite its use in water purification. In this study the antimicrobial properties of tungsten oxide are thought to be related to photocatalysis and hydrogen production. Studies have shown tungsten oxide to have photocatalytic activity in visible light [36]. However, it has also been reported that the band gap energy of tungsten oxide nanoparticles is much larger than the energy of visible light illumination, therefore the generation of reactive species (like ●OH radicals) is likely to be the result of native defects [37,38,39,40,41,42,43,44,45]. In the case of tungsten oxide, it can be said that the reduction in pathogenic microorganisms is the result of photokilling, whereby the reactive species damage the cell membrane causing the internal components to leak from the cells and are ultimately oxidised by the photocatalytic reaction [46,47,48,49,50].

For photocatalysts, with energy provided larger than the band gap, electron/hole pairs are generated and react with oxygen and water to form superoxide anion radicals (O_2_●^−^) and hydroxyl radicals (●OH) [49]. These oxidative species are highly reactive and considered to be the dominant oxidative species contributing to the death of bacterial cells [49].

The results obtained in the current study show that the inactivation of *S. aureus* using tungsten oxide nanoparticles in the presence of visible light is greater than that of *E. coli*. This observation indicates that Gram-positive bacteria are more susceptible to antimicrobial alternatives than that of Gram-negative species. This difference is commonly ascribed to the difference in cell wall structure. Gram-positive bacteria have a thick cell wall composed of many layers of peptidoglycan and teichoic acids whereas the cell wall of Gram-negative bacteria is relatively thin with an outer membrane containing lipopolysaccharides and lipoprotein bilayers [50]. In addition, the difference in potency may also be related to different affinities of microbial cell walls for tungsten oxide. Gram-negative bacteria are comparatively more resistant as their cell wall limits the absorption of many molecules and compounds. Consequently, a higher number of hydroxyl radical attacks for Gram-negative bacteria are needed for complete bacterial inactivation.

### 4.2. Antimicrobial Fibre Meshes

Nanocomposite fibres with 2, 4 and 8 wt % tungsten oxide embedded into a polymer matrix were successfully prepared through pressurised gyration. Despite the concentration of tungsten oxide, all fibres were continuous and bead-free, indicating that the intermolecular entanglement and chain overlap in the polymer solutions were sufficient to stabilise the polymer jet and create an adequate Rayleigh-Taylor instability for continuous fibre formation. This also confirms that the presence of tungsten oxide particles in the polymer solution did not greatly interfere with chain entanglement.

From the results, it was evident fibre diameter largely depended on tungsten oxide concentration. Increasing the concentration from 0 to 8 wt % led to the formation of thicker fibres with a wider fibre diameter distribution. This observation suggests the introduction of tungsten oxide particles causes the polymer suspension to have a greater resistance against the stretching caused by the forces on the polymer jet during novel gyration process. Previous studies claim fibre diameter can be tailored by altering working parameters such as polymer concentration in suspension, rotational speed and applied pressure [27,51]. However, during this experiment all working parameters were kept constant therefore suggesting tungsten oxide to be the underlying cause.

The antimicrobial activity of tungsten oxide/polymer nanocomposite fibres was assessed against *S. aureus* and bacteriophage T4 as tungsten oxide was shown to be potent towards these strains. The fibres were incubated in microbial suspensions for 24 h at 37 °C and 150 rpm.

All tungsten oxide/polymer fibres showed strong antiviral properties against bacteriophage T4, a DNA virus. DNA viruses such as adenovirus, pose a serious threat to human health. It is associated with a wide range of human illnesses, including respiratory, ocular, urinary tract and gastrointestinal diseases. Adenovirus is particularly problematic amongst the paediatric population, as it causes 2–8% of all childhood respiratory infections [4]. The ability of these fibres to reduce their existence in the environment, offers a promising solution.

The 2 wt % and 4 wt % tungsten oxide fibres showed no antibacterial activity when compared to the control (pure polymer fibres). At low concentrations, the presence of tungsten oxide nanoparticles on the surface of the fibres was relatively low. Therefore, the production and release of reactive oxygen species under visible light was limited. Since this concentration was determined to be the MIC of the fibres doped with nanoparticles, 2 wt % was chosen as the minimum tungsten oxide concentration in the fibres. Although there may be 2 wt % of tungsten oxide in the resulting fibre, not all of this would have been deposited on the fibre surface as most of them are trapped within the fibre. Therefore, increasing the tungsten oxide concentration further to 4 and 8 wt % increased the amount of tungsten oxide on the surface of the fibres.

When increasing the tungsten oxide concentration to 8 wt %, more of the nanoparticles were exposed on the surface of the fibres, as shown in Figure 5h,i,k,l [52]. This led to an antibacterial effect in which tungsten oxide destroyed the bacteria via a photocatalytic-killing mechanism. This was demonstrated and corroborated in Figure 7, as nonviable bacteria cells can be seen on the fibre surface.

Tungsten oxide/polymer nanocomposite fibres have presented themselves as a plausible way to reduce the number of health care acquired pathogens. This antimicrobial nanocomposite can be used in several applications in the healthcare setting, such as in filters and fabrics, to reduce the number of microbes in the environment. Reducing the number of pathogens by even 29% as a minimum requirement for the antimicrobial surface prevention materials will dramatically reduce the number of people that get infected and will consequently reduce the economic stress on the healthcare service.

## 5. Conclusions

The antimicrobial activity of tungsten, tungsten oxide and tungsten carbide were explored using Gram-negative and Gram-positive bacteria and a DNA virus. Tungsten oxide showed great potency towards Gram-positive bacteria (*S. aureus*) and the DNA virus (bacteriophage T4) at high concentrations (2.0 wt %), and therefore presented itself as a suitable antimicrobial agent. It is thought that tungsten oxide employs a photokilling mechanism of action against the microbes.

As the MIC of tungsten oxide was determined to be 2 wt %, polymeric fibres containing either 2, 4 or 8 wt % tungsten oxide were formed using pressurised gyration. The results obtained in this investigation indicated that fibre morphology was dependent on nanoparticle concentration. It was observed that as nanoparticle concentration increased, average fibre diameter increased. Average fibre diameter ranged between 1.5 ± 1.4 and 6.6 ± 4.4 µm.

Understanding the microbial activity of tungsten oxide/polymer nanocomposite fibres was critical. The effect of these fibres on the proliferation of *S. aureus* and *Escherichia* virus T4 were compared. Microbial studies revealed that 8 wt % of tungsten oxide was the MIC needed in the polymeric fibres to reduce microbial growth. Future studies involve investigating the effect nanoparticle size has on antimicrobial activity.

## Figures and Tables

**Table 1:** Tungsten oxide/polymer solution composition.

Nanoparticle Suspension		Polymer Solution		Final Concentration of Tungsten Oxide in the Resulting Fibre (wt %)
Tungsten Oxide Nanoparticle (g)	Chloroform (mL)	PMMA (g)	Chloroform (mL)	
0.00	10	4	10	0
0.08	10	4	10	2
0.16	10	4	10	4
0.32	10	4	10	8

## Supplementary Materials

The following are available online at https://www.mdpi.com/2079-4991/10/6/1017/s1, Figure S1: Histograms showing the diameter distribution of the formed fibres: (a) pure PMMA fibres; (b) PMMA fibres with 2 wt % WO_3_; (c) PMMA fibres with 4 wt % WO_3_; (d) PMMA fibres with 8 wt % of WO_3_.
