# Supplementary material for: Comparative Study of the Antimicrobial Effects of Tungsten Nanoparticles and Tungsten Nanocomposite Fibres on Hospital Acquired Bacterial and Viral Pathogens
Source: Nanomaterials (Basel). 2020 May 26;10(6):1017. doi: 10.3390/nano10061017 (PMC7352352; doi:10.3390/nano10061017)
Supplement: Supplementary file 1 [file nanomaterials-10-01017-s001.pdf]

nanomaterials

Supplementary Material

Comparative Study of the Antimicrobial Effects of Tungsten Nanoparticles and Tungsten Nanocomposite Fibres on Hospital Acquired Bacterial and Viral Pathogens

Rupy Kaur Matharu [1,2], Lena Ciric [2], Guogang Ren [3] and Mohan Edirisinghe [1,*]

[1] Department of Mechanical Engineering, University College London, Torrington Place, London, WC1E 7JE, UK; rupy.matharu.15@ucl.ac.uk
[2] Department of Civil, Environmental & Geomatic Engineering, University College London, Chadwick Building, Gower Street, London, WC1E 6BT, UK; l.ciric@ucl.ac.uk
[3] School of Engineering and Technology, University of Hertfordshire, Hatfield AL10 9AB, UK; g.g.ren@herts.ac.uk
* Correspondence: m.edirisinghe@ucl.ac.uk

nanomaterials

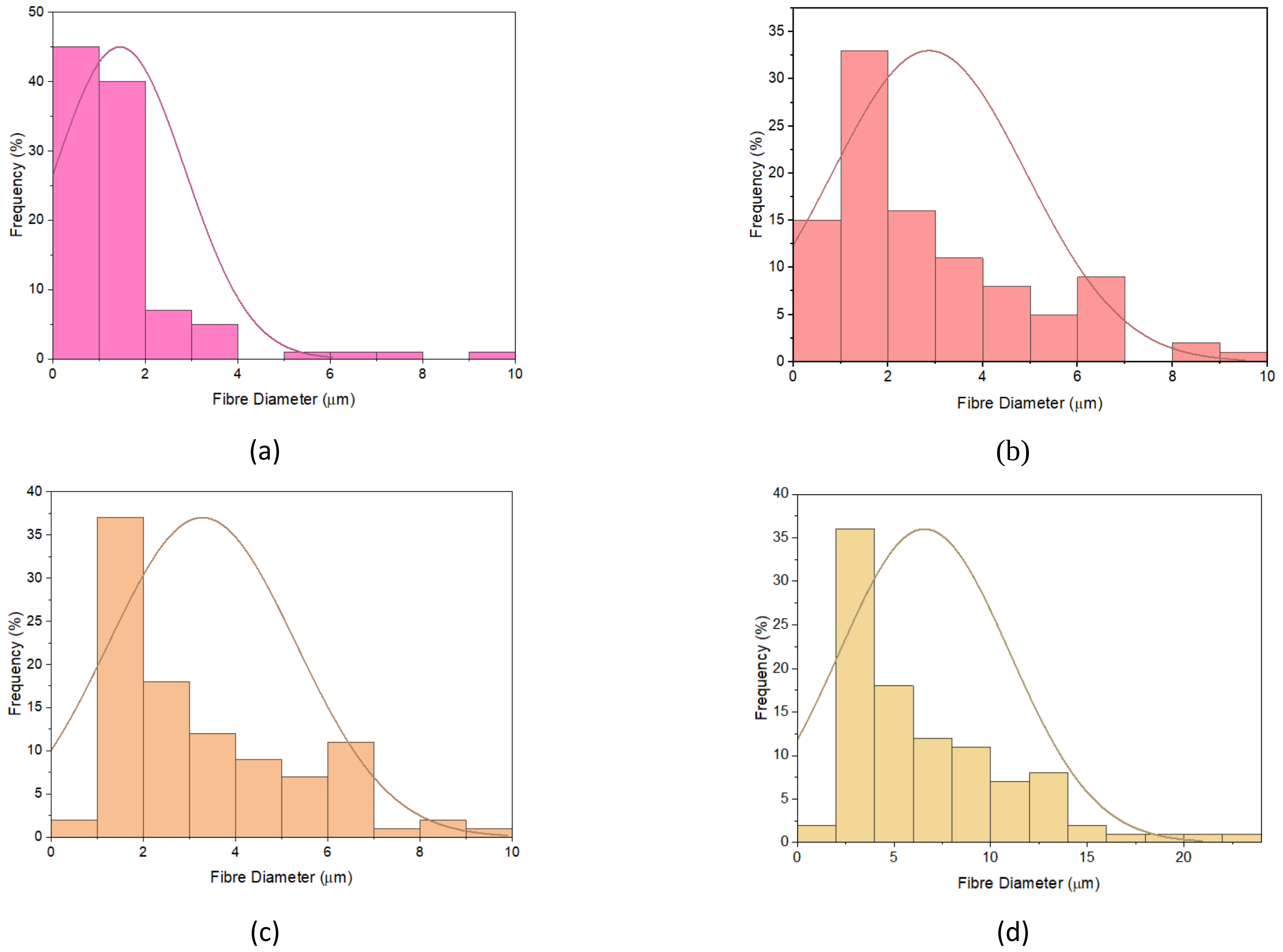

Figure S1. Histograms showing the diameter distribution of the formed fibres: (a) pure PMMA fibres; (b) PMMA fibres with 2 wt % WO_3; (c) PMMA fibres with 4 wt % WO_3; (d) PMMA fibres with 8 wt % of WO_3.

© 2020 by the authors. Submitted for possible open access publication under the terms and conditions of the Creative Commons Attribution (CC BY) license (http://creativecommons.org/licenses/by/4.0/).
